# OLDER ADULTS’ INTERACTIONS ON SHORT-FORM VIDEO PLATFORMS ENHANCE THEIR SOCIAL ADAPTATION: A CHAIN MEDIATION MODEL

**DOI:** 10.1093/geroni/igae098.3505

**Published:** 2024-12-31

**Authors:** Jingxuan Wu, Huamao Peng

**Affiliations:** Beijing Normal University, Beijing, Beijing, China (People’s Republic); Beijing Normal University, Beijing, Beijing, China (People’s Republic)

## Abstract

**Background:**

An increasing number of older adults are embracing short-form video platforms like TikTok. Beyond passive watching, how active engagement by older adults on short-form video platforms influences their social adaptation deserves significant attention. Objective: Explore the effect of interactions on short-form video platforms (e.g., liking, commenting, sharing, and rewarding, etc.) on social adaptation among older adults and examine the chain mediating roles of basic psychological needs satisfaction (competence, autonomy, and relatedness) and attitudes towards self ageing to reveal the underlying psychological mechanism.

**Method:**

Two thousand older adults (aged 55–85 years old, Mage = 60.20±3.88) completed the Social Adaptation Self-evaluation Scale, the Short-form Video Interactive Behavior Questionnaire, the Basic Psychological Needs Scale, and the Attitudes to Ageing Questionnaire. PROCESS Model 6 was used to test a chain multiple mediation model.

**Results:**

Frequent short-form video interactions of older adults not only directly influence their social adaptation level positively but also indirectly contribute to its improvement through more satisfaction of basic psychological needs and greater positive attitudes towards self ageing.

**Discussion:**

More interactions on short-form videos platforms contribute to the satisfaction of older adults’ basic psychological needs. This, in turn, encourages a comparatively positive attitude towards ageing, and ultimately bolstering adaptability across various aspects of later life. This insight motivates older adults to immerse themselves more deeply and actively participate in short-form videos, utilizing the new media application as an emerging space for social engagement, thereby accumulating psychosocial well-being to achieve active and successful aging.

